# Turnover of Sex Chromosomes in *Celebensis* Group Medaka Fishes

**DOI:** 10.1534/g3.115.021543

**Published:** 2015-10-23

**Authors:** Taijun Myosho, Yusuke Takehana, Satoshi Hamaguchi, Mitsuru Sakaizumi

**Affiliations:** * Institute of Science and Technology, Niigata University, Niigata 950-2181, Japan; † Laboratory of Bioresources, National Institute for Basic Biology, Okazaki 444-8585, Japan

**Keywords:** sex-determining gene, convergent evolution, sex chromosome, *Sox*, medaka, genetics of sex

## Abstract

Sex chromosomes and the sex-determining (SD) gene are variable in vertebrates. In particular, medaka fishes in the genus *Oryzias* show an extremely large diversity in sex chromosomes and the SD gene, providing a good model to study the evolutionary process by which they turnover. Here, we investigated the sex determination system and sex chromosomes in six *celebensis* group species. Our sex-linkage analysis demonstrated that all species had an XX-XY sex determination system, and that the *Oryzias marmoratus* and *O. profundicola* sex chromosomes were homologous to *O. latipes* linkage group (LG) 10, while those of the other four species, *O. celebensis*, *O. matanensis*, *O. wolasi*, and *O. woworae*, were homologous to *O. latipes* LG 24. The phylogenetic relationship suggested a turnover of the sex chromosomes from *O. latipes* LG 24 to LG 10 within this group. Six sex-linkage maps showed that the former two and the latter four species shared a common SD locus, respectively, suggesting that the LG 24 acquired the SD function in a common ancestor of the *celebensis* group, and that the LG 10 SD function appeared in a common ancestor of *O. marmoratus* and *O. profundicola* after the divergence of *O. matanensis*. Additionally, fine mapping and association analysis in the former two species revealed that *Sox3* on the Y chromosome is a prime candidate for the SD gene, and that the Y-specific 430-bp insertion might be involved in its SD function.

Sex determination mechanisms in animals are extremely diverse. Most vertebrates have genetic sex determination with sex chromosomes, while their origins differ among taxa. Such turnover of sex chromosomes is associated with the substitution or translocation of master sex-determining (SD) genes. To date, nine SD genes or candidates have been identified on different sex chromosomes in mammals, birds, frogs, and fishes (Gubbay *et al.* 1990; Sinclair *et al.* 1990; Matsuda *et al.* 2002; Yoshimoto *et al.* 2008; Smith *et al.* 2009; Hattori *et al.* 2012; Kamiya *et al.* 2012; Myosho *et al.* 2012; Yano *et al.* 2012; Takehana *et al.* 2014), confirming that different SD genes have contributed to the development of new sex chromosomes during vertebrate evolution. These studies also suggest that several groups of genes tend to be recruited as the master SD signals, because five of them belong to the transcription factor genes (*Sox3*-related or *Dmrt1*-related) and other three are involved in the TGF-beta signaling pathway (*Amh*, *Amhr2*, and *Gsdf*). To understand the limited diversity of SD genes, we must first elucidate a common mechanism of SD gene emergence by identifying the molecular mechanism by which each gene arose. However, most of the above vertebrate species whose SD genes have been identified are distantly related to each other, which makes tracing the evolutionary process of SD gene turnover by comparing them difficult. However, it may be easier in closely related species that have common genes and pathways involved in sex determination because they share recent common ancestors. In some fish groups, such as salmonids and sticklebacks, high rates of sex chromosome turnover have been documented among closely related species (Ross *et al.* 2009; Woram *et al.* 2003). Among them, the SD genes have not been uncovered in sticklebacks, and the majority of salmonids share the same SD gene, *sdY*, which has potentially jumped into different ancestral autosomes (Yano *et al.* 2013).

Medaka fishes in the genus *Oryzias* show an amazingly large diversity in their sex determination systems and sex chromosomes, providing an excellent model group for investigating the molecular mechanisms underlying the rapid turnover of sex chromosomes. They possess both XY and ZW systems, and their sex chromosomes differ from one species to another (Tanaka *et al.* 2007; Takehana *et al.* 2007a, 2007b, 2008; Nagai *et al.* 2008). Among the eight species analyzed to date, only three (*Oryzias latipes*, *O. sakaizumii*, and *O. curvinotus*) share the same sex determination mechanism (Figure 1; Matsuda *et al.* 2002, 2003). In these species, *Dmy* on the homologous Y chromosome is the SD gene, but this gene has not been found in other congeneric species. Instead, different SD genes have been isolated; *Gsdf^Y^* in *O. luzonensis* (Myosho *et al.* 2012) and *Sox3^Y^* in *O. dancena* (Takehana *et al.* 2014), suggesting that different sex chromosomes have evolved through the acquisition of new SD genes. However, all of these species are members of the *latipes* and *javanicus* groups in *Oryzias*, and no information is available on the members of the remaining *celebensis* group.

**Figure 1:**
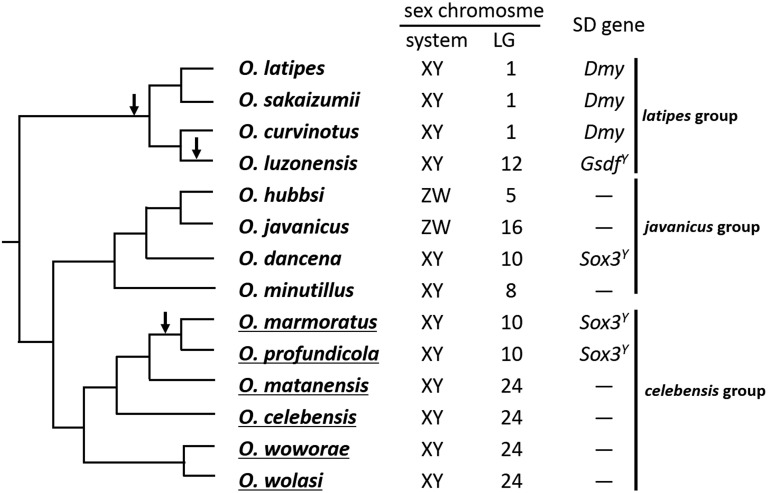
Phylogenetic relationships and sex determination in *Oryzias* fishes. The phylogenetic information was taken from Takehana *et al.* (2005) and Mokodongan and Yamahira (2015). Arrows indicate nodes at which the sex chromosome turnovers occurred. Species examined in this study are underlined.

All species in the *celebensis* group are endemic to Sulawesi Island, Indonesia. To date, 14 described species—approximately half of the species in the genus—are endemic to this island, suggesting that Sulawesi is a diversity hotspot for the medaka fishes. Previous molecular phylogenetic analysis has revealed that the *celebensis* group is monophyletic, and genetic divergences among the species are relatively lower than those in other groups (Takehana *et al.* 2005). In this study, we isolated sex-linked DNA markers and identified the sex chromosomes of six species in the *celebensis* group. All species had an XY sex determination system, but their sex chromosomes differed: those of *O. marmoratus* and *O. profundicola* were homologous to *O. latipes* LG 10, while those of *O. celebensis*, *O. matanensis*, *O. wolasi*, and *O. woworae* were homologous to *O. latipes* LG 24. We also identified possible SD gene candidates on these sex chromosomes. In particular, the *O. marmoratus* and *O. profundicola* SD loci were tightly linked to the *Sox3* gene, which is also the SD gene in *O. dancena* from the *javanicus* group, suggesting that it has been independently recruited as the SD gene in different lineages of this genus.

## Materials and Methods

### Fish

*O. celebensis*, *O. marmoratus*, *O. matanensis*, and *O. profundicola* were supplied by Niigata University, a subcenter of the National BioResource Project (Medaka) (http://www.shigen.nig.ac.jp/medaka/). These species were originally collected at Ujung Pandang, Lake Towuti, Lake Matano, and Lake Towuti in Sulawesi, Indonesia, respectively. *O. wolasi* and *O. woworae* were obtained commercially from aquarium shops in Japan. Their collection localities are unknown, but their original distributions are southeastern Sulawesi (Parenti and Hadiaty 2010; Parenti *et al.* 2013). All fish were raised and maintained at 27 ± 2° under a 14:10 h light:dark cycle.

### Genetic crosses, sexing, and DNA extraction

In each species, one female and one male were crossed to generate their F1 progeny. Single pairs of the progeny were subsequently intercrossed to obtain F2 and F3 generations. We obtained 361 progeny (72 F1, 153 F2, and 136 F3) in *O. celebensis*, 181 (54 F1 and 127 F2) in *O. marmoratus*, 83 F1 in *O. matanensis*, 78 F1 in *O. profundicola*, 201 (32 F1 and 169 F2) in *O. wolasi*, and 66 F1 in *O. woworae*. Phenotypic sex was determined by adult fish secondary sex characteristics, namely, the shapes of dorsal and anal fins. Total DNA was extracted from the caudal fin by proteinase K digestion, phenol-chloroform extraction, and isopropanol precipitation (Shinomiya *et al.* 1999). DNA samples were dissolved in TE buffer.

### Isolation of sex linked markers and linkage analysis

To identify polymorphisms between the male and female parents, we screened expressed sequence tag (EST) markers developed in *O. latipes* (http://mbase.nig.ac.jp/mbase/medaka_top.html) (Naruse *et al.* 2004). As for the markers on LG 10 and LG 24, we designed new STS primers on conserved exons across introns based on the medaka and stickleback genomic sequences (http://asia.ensembl.org/index.html) (Supporting Information, Table S1). ESTs and STSs were amplified under the following touchdown PCR conditions: 5 min at 95°; 12 cycles of 30 s at 95°, 30 s at 56–65° (−3° for every three cycles), 2 min at 72°; 22 cycles of 30 s at 95°, 30 s at 53°, 2 min at 72°; and 2 min at 72°. Polymorphisms in each amplified marker were analyzed by polyacrylamide gel electrophoresis to detect insertion, deletion, heteroduplex, and PCR-RFLPs. Sex-linkage maps were constructed for the polymorphic markers in all six species.

### SD region sequencing in *O. marmoratus* and *O. profundicola*

We designed original primers based on the consensus regions between *O. latipes* (http://asia.ensembl.org/index.html) and *O. dancena* (accession numbers: AB909496 and AB909497) LG 10 sequences, and determined the DNA sequences of *O. marmoratus* by PCR direct sequencing and sequencing after cloning of the PCR products. We used an XX female and an XY male to determine the X and Y chromosome sequences. The X and Y sequences were deposited in GenBank under the accession numbers LC037173–LC037176. We also determined partial *O. profundicola* X and Y chromosome sequences to check 55 Y-specific mutation sites found in the *O. marmoratus* SD region.

### Association analysis

We obtained wild *O. marmoratus* (*N* = 68) and *O. profundicola* (*N* = 34) stocks from Shinshu University and the National Institute for Basic Biology. We genotyped 10 Y-specific mutation regions conserved between the *O. marmoratus* and *O. profundicola* Y chromosomes by PCR-direct sequencing. Primer sequences for genotyping were as follows: the #1 and #2 common Y-specific mutations, CGGATTGAATGTCGTTTTCC and TGACCTCATTCCCGGAAAGGA; #3, TTTTTGATTGAAAAGAAGAGAGTTT and ACATGCATTTCACCCCTCTC; #4 and #5, TCTGTTGAAGGTCTGGGTCTC and TTTACGCACGCTCTGTTGTC; #6–9, TTCCGAGTTCAAAACTCTTGC and CCAAACGTTGGCTTCAAATC; #10, GTACCCCCACATCCTGTCAC and TTATTACCAGTGGGGCAACG.

## Results

**Figure 2:**
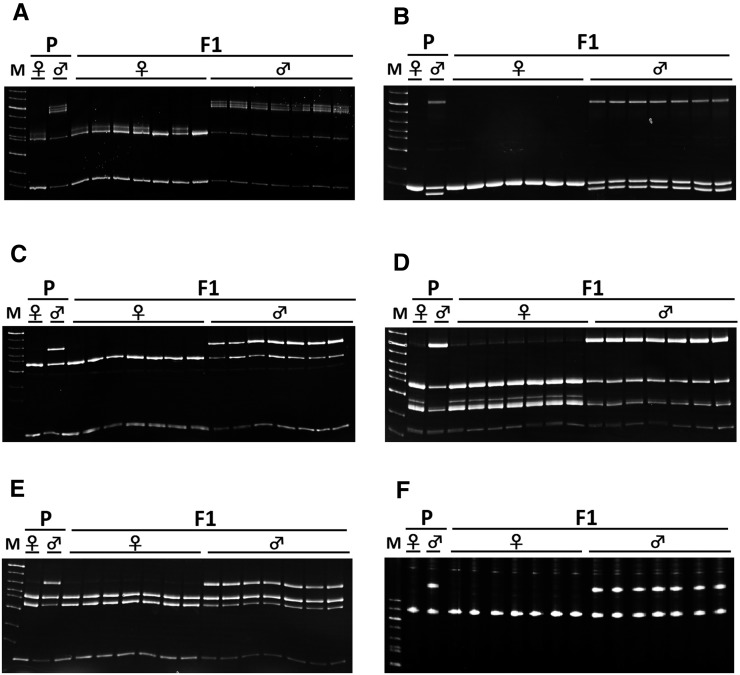
Male heterogametic inheritance of sex-linked markers in *celebensis* group *Oryzias* fishes. Electrophoretic patterns of SOX3 PCR product digested with *Mse*I in *O. marmoratus* (A), MF01SSA075C05 PCR product in *O. profundicola* (B), PCMT1 digested with *Bsm*AI in *O. celebensis* (C), HNRPU digested with *Hin*dIII in *O. matanensis* (D), PGM digested with *Hph*I in *O. wolasi* (E), and SOX7 in *O. woworae* (F). Note that the male-specific bands in the parents segregated perfectly with F1 males. F1, F1 progeny from the parents; M, marker; P, parents.

To isolate sex-linked markers in six species, *O. celebensis*, *O. marmoratus*, *O. matanensis*, *O. profundicola*, *O. wolasi*, and *O. woworae*, we screened over 250 ESTs established in *O. latipes*. We detected polymorphic patterns in the EST markers between the parents, and assessed whether or not the polymorphisms cosegregated with the phenotypic sex. We also designed and genotyped new STS markers on the same chromosome as the ESTs to obtain additional sex-linked markers. As a result, we successfully isolated sex-linked markers from all six species, namely five markers from *O. celebensis*, nine from *O. marmoratus*, six from *O. matanensis*, five from *O. profundicola*, five from *O. wolasi*, and four from *O. woworae*. For the completely sex-linked markers, all male progeny had the paternal genotype, while all female progeny had the maternal genotype, indicating that all of these species had an XX-XY sex determination system (Figure 2).

**Figure 3:**
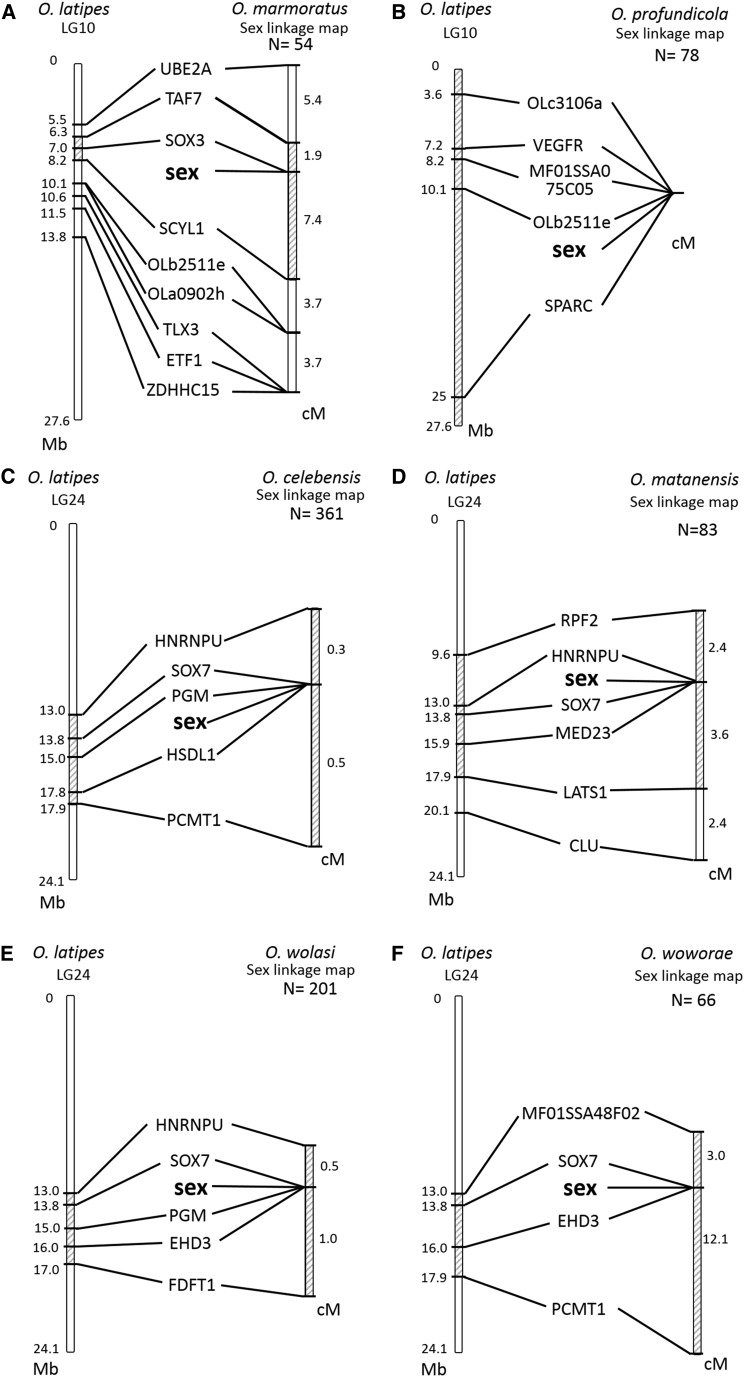
Sex-linkage maps of DNA markers in *O. marmoratus* (A), *O. profundicola* (B), *O. celebensis* (C), *O. matanensis* (D), *O. wolasi* (E), and *O. woworae* (F). Comparison of the gene orders between the sex-linkage map in six species and the physical *O. latipes* LG 10 and LG 24 maps are also shown. Lines between the compared chromosomes connect the positions of orthologous gene pairs. The distances between flanking markers are shown as either physical length or map distance. The shaded area represents the sex-determining (SD) regions on the genetic maps and the corresponding regions on the physical maps.

We constructed sex-linkage maps using the markers, and identified the SD loci in all six species (Figure 3). All of the *O. marmoratus* and *O. profundicola* sex-linked markers were located on *O. latipes* LG 10, while those of *O. celebensis*, *O. matanensis*, *O. wolasi*, and *O. woworae* were on *O. latipes* LG 24. Gene orders were conserved between the sex-linkage maps and the physical *O. latipes* maps, indicating conserved syntenies around the SD locus between the species. These results suggest that the sex *O. marmoratus* and *O. profundicola* chromosomes are homologous to *O. latipes* LG 10, and those of *O. celebensis*, *O. matanensis*, *O. wolasi*, and *O. woworae* to *O. latipes* LG 24. Furthermore, the mapped SD loci positions were consistent between *O. marmoratus* and *O. profundicola* and among *O. celebensis*, *O. matanensis*, *O. wolasi*, and *O. woworae*, suggesting single origins of both types of sex chromosome.

To elucidate the exact location of the SD locus in *O. marmoratus*, we performed further linkage analysis by adding 127 F2 progeny (in total *N* = 181). This analysis pinpointed the SD locus within a 1.4 cM region between two STS markers: SOX3-up and SOX3-down. This region was approximately 34.4 kb in *O. latipes*, and contained only one gene, *Sox3*, based on its draft genome sequence data (Figure 3A). We then determined entire nucleotide sequences for the *O. marmoratus* SD region, and found that the X and Y sequences were 29.8 kb and 30.2 kb in length, respectively. Both SD regions contained only the *Sox3* gene, suggesting this as a prime candidate for the SD gene in *O. marmoratus*.

**Figure 4:**
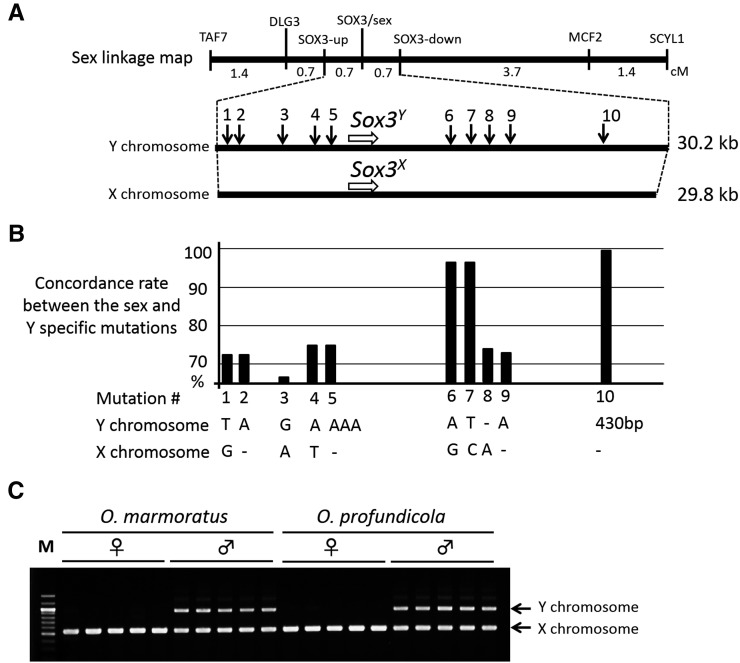
Positional cloning and association analysis of the SD locus on the sex chromosomes shared between *O. marmoratus* and *O. profundicola*. (A) A high resolution genetic map around the *O. marmoratus* SD region using 181 progeny (top), and physical maps of the region on the Y and X chromosomes (bottom). The SD locus is mapped within a genetic interval (1.4 cM) between two markers (SOX3-up and SOX3-down), and completely links to the marker SOX3. Arrows indicate Y-specific mutations common to *O. marmoratus* and *O. profundicola*. (B) Association analysis between the phenotypic sex and Y-specific mutations in *O. marmoratus* and *O. profundicola*. Only the 430-bp insertion perfectly matches phenotypic sex in *O. marmoratus* (*N* = 68) and *O. profundicola* (*N* = 34). (C) Electrophoretic pattern of PCR product including the 430-bp insertion in *O. marmoratus* and *O. profundicola*. Upper bands indicate the Y chromosome alleles having the insertion. Lower bands indicate the X chromosome alleles without the insertion.

No mutations in the *Sox3* gene coding sequences suggested that its regulatory mutations might contribute to the sex-determining function, as in the case of *Sox3^Y^* in *O. dancena* (Takehana *et al.* 2014). To identify the responsible sequences in the SD region, we compared the sequences between the *O. marmoratus* X and Y chromosomes, and detected 55 Y-specific mutations, comprising 37 SNPs and 18 indels. To find conserved mutations between species, we checked these mutation sites on the *O. profundicola* X and Y chromosomes, because this species was assumed to have the same SD locus. By comparing X and Y chromosome sequences between the two species, we successfully identified ten common Y-specific mutations, five SNPs and five indels (Figure 4A). Further association analysis using wild *O. marmoratus* (*N* = 68) and *O. profundicola* (*N* = 34) stocks revealed that they only had a single 430-bp insertion Y chromosome-specific mutation in common (Figure 4, B and C). This insertion was not homologous to other sequences deposited in the public database, and was not found in the *O. dancena* SD region, suggesting that it might contribute to the *Sox3* SD function in *O. marmoratus* and *O. profundicola*.

## Discussion

The present study clearly demonstrated the sex determination system, sex chromosomes, and SD loci in six *celebensis* group medaka fishes. All sex-linked markers isolated showed male-heterogametic inheritances, indicating that all six species have an XX-XY sex determination system. Previous studies have revealed that all four species, in the *latipes* group (Matsuda *et al.* 2003; Hamaguchi *et al.* 2004), and two species, *O. dancena* and *O. minutillus*, in the *javanicus* group have an XX-XY sex determination system (Takehana *et al.* 2007a; Nagai *et al.* 2008). However, two species, *O. hubbsi* and *O. javanicus*, in the *javanicus* group have a ZZ-ZW system (Takehana *et al.* 2007b, 2008). Therefore, 12 of the 14 *Oryzias* species have an XX-XY sex determination system, suggesting that the XX-XY system is dominant in this genus.

Sex LGs have been identified in eight *Oryzias* species; namely *O. latipes* and *O. sakaizumii* (Naruse *et al.* 2000), *O. curvinotus* (Matsuda *et al.* 2003), *O. luzonensis* (Tanaka *et al.* 2007), *O. minutillus* (Nagai *et al.* 2008), *O. dancena* (Takehana *et al.* 2007a), *O. hubbsi* (Takehana *et al.* 2007b), and *O. javanicus* (Takehana *et al.* 2008). The sex chromosomes differed from species to species, with the exception of *O. latipes*, *O. sakaizumii*, and *O. curvinotus*, which have the same SD gene *Dmy* (see Figure 1). In the present study, we successfully identified the sex LG in the six *celebensis* group species. The *O. marmoratus* and *O. profundicola* sex chromosomes were homologous to *O. latipes* LG 10, while those of *O. celebensis*, *O. matanensis*, *O. wolasi*, and *O. woworae* were homologous to *O. latipes* LG 24. Sex chromosomes of LG 10 and 24 were considered to share the same SD genes, respectively, because the positions of the SD loci were conserved between the species.

Although *O. marmoratus* and *O. profundicola* shared the same sex chromosomes, their sex-linkage maps differed in terms of mapping distance. In the *O. marmoratus* sex-linkage map, we identified a small SD region because of frequent recombination. In contrast, no recombination (0/78) was observed in the *O. profundicola* sex chromosome pair, suggesting that it might be suppressed along these chromosomes in this species. Similar situations were also observed in the *O. celebensis*, *O. matanensis*, *O. wolasi*, and *O. woworae* sex-linkage maps. Their sex-linked markers clustered around the SD loci, 0/361 recombination in *O. celebensis*, 0/83 in *O. matanensis*, 0/200 in *O. wolasi*, and 0/66 in *O. woworae*. This recombination suppression is likely associated with chromosome rearrangements, such as an inversion around the SD locus. The absence of recombination associated with chromosome rearrangements has been reported in other *Oryzias* species (Takehana *et al.* 2007b) and sticklebacks (Peichel *et al.* 2004; Ross and Peichel 2008). However, Matsuda *et al.* (1999) reported that maleness rather than heterogametic sex chromosome suppresses recombination in *O. latipes*. To test two possibilities, we need to examine the sex chromosome structure by FISH analysis and the recombination frequency in sex-reversed females.

We have successfully identified *Sox3* as a prime candidate for the SD gene in *O. marmoratus* and probably in *O. profundicola*. This is the sole gene in the *O. marmoratus* SD region, and the 430-bp Y-specific insertion is associated with phenotypic sex in wild stocks of these species, suggesting that the insertion might contribute to its male-determining function. In another *Oryzias* species, *O. dancena*, it has been shown that the *Sox3* gene on the Y chromosome is required and sufficient in male determination (Takehana *et al.* 2014). However, the 430-bp insertion was not found in the *O. dancena* SD region, suggesting that these *Sox3*-dependent sex determination mechanisms have evolved independently. Furthermore, the mammalian SD gene, *Sry*, is considered to have evolved from *Sox3* as an allelic variant (Foster and Graves 1994; Sutton *et al.* 2011). Thus, the independent recruitments of *Sox3* to male determination appears to have occurred at least three times in vertebrates.

In *O. celebensis*, *O. matanensis*, *O. wolasi*, and *O. woworae*, we identified the common SD loci on their sex chromosomes, which were homologous to *O. latipes* LG 24. Using the Ensemble genome browser, we identified 125 protein-coding genes within the region ranging from 13.0 to 17.0 Mb of *O. latipes* LG 24, which corresponded to their SD loci. There is no well-known sex-related gene among these genes, except for *Sox7*. Our linkage analysis showed that this gene was tightly linked to phenotypic sex in all four species. More than 20 *Sox* family genes have been identified so far, and classified into nine groups based on their sequence homology (Bowles *et al.* 2000). Of these, seven genes (*Sry*, *Sox3*, *Sox8*, *Sox9*, *Sox10*, *Sox15*, and *Sox17*) are involved in either mammalian sex determination or differentiation, suggesting that these *Sox* genes potentially have an SD function (Koopman *et al.* 1991; Sutton *et al.* 2011; Barrionuevo *et al.* 2009; Chaboissier *et al.* 2004; Polanco *et al.* 2010; Sarraj *et al.* 2003; Kanai *et al.* 1996). Further analysis of the SD function of *Sox7* in the *Oryzias* species is required.

In the *celebensis* group, the basal lineages shared the same SD loci, suggesting that the common ancestor of this group had sex chromosomes homologous to *O. latipes* LG 24. Thus, a transition of the different sex determination mechanisms likely occurred in the common ancestor of the sister species pair, *O. marmoratus* and *O. profundicola*, after the divergence of *O. matanensis*, because they share the common SD loci on the sex chromosomes that were homologous to *O. latipes* LG 10. A recent molecular phylogenetic study reconfirmed the close genetic relationship between *O. marmoratus*, *O. profundicola*, and *O. matanensis*, and estimated their divergence time at less than 2.5 million years ago (Mokodongan and Yamahira 2015). Additionally, these three species inhabit in the same Malili Lake System in central Sulawesi, the former two species in Lake Towuti and the latter in Lake Matano (Kottelat 1990). Thus, these findings suggest that the turnover of the sex chromosomes occurred very recently in these species, likely during the speciation process in in the different lakes.

## Supplementary Material

Supporting Information
