# Supplementary material for: Turnover of Sex Chromosomes in Celebensis Group Medaka Fishes
Source: G3 (Bethesda). 2015 Oct 23;5(12):2685–91. doi: 10.1534/g3.115.021543 (PMC4683641; doi:10.1534/g3.115.021543)
Supplement: Supporting Information [file supp_5_12_2685__index.html]

Turnover of Sex Chromosomes in Celebensis Group Medaka Fishes — Supporting Information 

# Turnover of Sex Chromosomes in *Celebensis* Group Medaka Fishes

## Supporting Information for Myosho *et al.*, 2015

**Files in this Data Supplement:**

- Table S1 - EST and STS primers used in this study. (.xlsx, 13 KB)
